# Macrofungal species distributions depend on habitat partitioning of topography, light, and vegetation in a temperate mountain forest

**DOI:** 10.1038/s41598-018-31795-7

**Published:** 2018-09-11

**Authors:** Yun Chen, Zhiliang Yuan, Shuai Bi, Xueying Wang, Yongzhong Ye, Jens-Christian Svenning

**Affiliations:** 1 College of Life Sciences, Henan Agricultural University, No.63 Agricultural Road, Zhengzhou, 450002 China; 2 Section for Ecoinformatics and Biodiversity, Department of Bioscience, Aarhus University, Aarhus, Denmark; 3 Center for Biodiversity Dynamics in a Changing World (BIOCHANGE), Aarhus University, Aarhus, Denmark

## Abstract

The habitat partitioning hypothesis provides a conceptual framework for explaining the maintenance of plant and animal diversity. Its central tenet assumes environmental conditions are spatially structured, and that this structure is reflected in species distributions through associations with different habitats. Studies confirming habitat partitioning effects have focused primarily on spatial distributions of plants and animals, with habitat partitioning hypothesis under explored for macrofungi. Here, we examined the sporocarps of macrofungi in a 5-ha forest dynamics plot in China. We used four different methods to define microhabitats for habitat partitioning analyses based on topography, understory light availability, plant community, or a combination of these factors, and analyzed the effect of microhabitat partitioning on epigeous macrofungal community. Our results showed that the characteristics of the macrofungal assemblages varied among the habitats. A total of 85 species examined were associated with one or more of the habitat types (85/125, 68%). The factors related to the sporocarp composition differed among the various microhabitats. Our findings suggest that different microhabitats favor occurrence of different macrofungal species, and sporocarps -environment relation varied among the different microhabitats at this temperate mountain forest locality. These findings shed new light to the biodiversity conservation in macrofungi in temperate deciduous broad-leaved forest and point to the potential importance of microhabitat partitioning for sporocarp formation.

## Introduction

Macrofungi – fungi with a fruiting body observable by the naked eye^1^ – are important components in biodiversity and the primary agents of decomposition in natural ecosystems^2–5^. Macrofungi are important in the cycling of various elements, such as carbon, nitrogen, and oxygen^1^. Despite their ecological importance, the mechanism driving macrofungal community assembly is not as well-known as those in plants and animals^6^.

Local species composition and spatial distributions are often associated with environmental factors (e.g., topography, soil, and light)^7–11^. According to niche theory, species coexistence can be attributed to species differences in environmental requirements and the distribution of environmental conditions in space, also by interspecific competition and facilitation^12,13^. If niche-relevant environmental conditions are spatially structured, then they should be reflected in species distributions through associations of species with different microhabitats^14,15^. Consequently, distinct species assemblages should be formed at community-level among different microhabitats^14,15^. Previous studies have reported that many species in forest ecosystems have distinct specific microhabitat preferences and have highlighted the importance of microhabitat characteristics for defining species assemblages^8,9,11,16^. However, these studies have focused on plants^8,9,11,14,16–18^, while the role of microhabitat partitioning for macrofungal community assembly remains poorly known.

Topography is considered an important driver of microhabitat diversity in forest ecosystems^14,19^. Various microhabitat types may be defined by topographic features^8,9,14,16–18^, with ridge and valley habitats the most common microhabitats associated with distinct species communities^20^. However, unlike light or soil properties, topography is an indirect environmental variable, without direct biological effects^18^, but ridges and valleys may differ in soil, light, and microclimatic variables, all factors which may affect fungal communities and potentially also shift relationships between fungi and environment^20^. For example, sporocarp growth has been related to light^21,22^, and development of primordia of fruiting bodies of many macrofungi is triggered by light^1^. In addition, plant community composition determines understory light availability, humidity and litter composition^21–23^. At same time, many macrofungal species have host associations to certain plant species, and the planty community therefore constitutes a biotic factor of key importance for fungal composition^24–26^. Previous studies of microhabitat partitioning in plants and animals have been emphasized the importance of topography^18,20^, while the roles of understory light availability and vegetation for species microhabitat partitioning remains ambiguous. Furthermore, the characteristics of the macrofungal assemblages, and their relationships with the environment in different microhabitats in forest ecosystems have not been elucidated.

Temperate mountain forests are widely distributed in northern China^27^ and harbor a high diversity of macrofungal species^1^. These forests have wide variations in topographic, light, and plant conditions, which presumably provide niches to accommodate diverse macrofungi. However, human interference and environmental damage resulting in decrease of number and diversity of macrofungi is a widespread phenomenon in temperate mountain forests in China^1^, with some macrofungal species even at risk of extinction^5^. Conservation awareness and measures of macrofungal resources is largely lacking^5^. Elucidating the community assembly mechanism in macrofungi is a crucial step to conserve their diversity.

In the present study, we hypothesized that microhabitat specialization is important for structuring epigeous macrofungal communities at small spatial scales in temperate mountain forest ecosystems, and hence would be important for the maintenance of their local species diversity. Multivariate regression tree (MRT) was computed to delineate microhabitat types for epigeous macrofungal communities. To test these hypotheses, we examined microhabitat preferences of macrofungal species by Indicator Species Analysis and Torus-translation test, and then analyzed the sporocarp-environment relations among different forest microhabitats by Mantel tests and multiple regressions of distance matrices (MRM) models.

## Results

### Delineation of microhabitats for epigeous macrofungal communities

**Figure 1:**
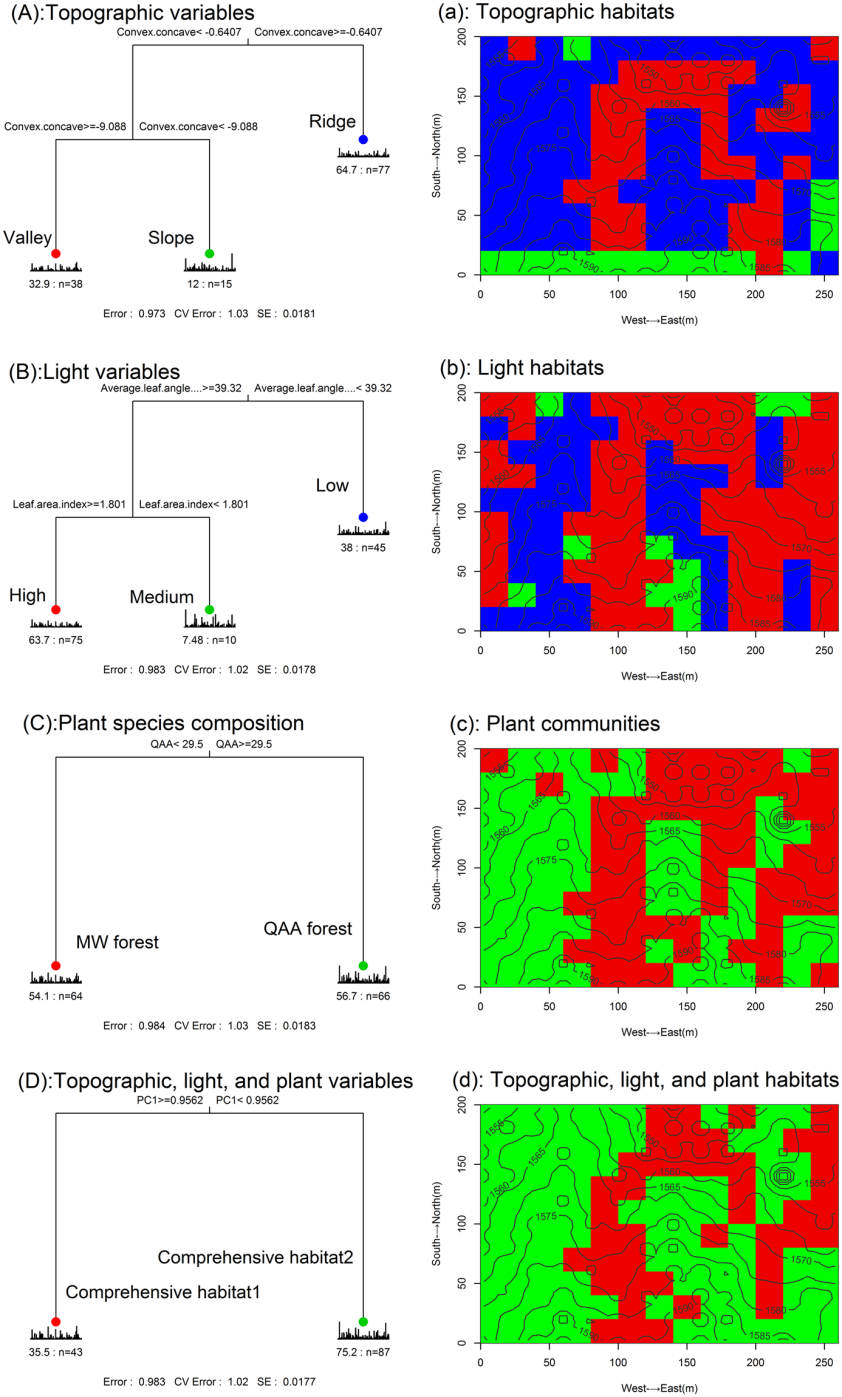
Results of multiple regression tree analysis of the sporocarp composition with topographic variables ((**A**) and a: red is valley; green is slope, and blue is ridge.), light variables ((**B**) and b: red is high; green is medium, and blue is low.), plant species composition ((**C**) and c: red is MW forest; green is QAA forest.), and comprehensive habitats ((**D**) and d: red is habitat1; green is habitats2). MW forest: Mixed wood forest; QAA forest: *Quercus aliena* var. *acuteserrata* forest. Comprehensive habitats are delineated based on the principal component analysis of convex concave, leaf area index, average leaf angle, and species abundance of *Quercus aliena* var. *acuteserrata*.

The multivariate regression tree analysis classified the Baiyunshan plot into valley (n = 38), slope (n = 15), or ridge (n = 77) habitat types, according to their convex concave degree. High-light (n = 75), medium-light (n = 10), or low-light (n = 45) light habitat types were separated by leaf area index and average leaf angle. Mixed wood forest (MW) (n = 64) and *Quercus aliena* var. *acuteserrata* forest (QAA) (n = 66) were separated by species abundance of *Q. aliena* var. *acuteserrata*. The results showed that there were some similarities in the classification of these three habitat types (topographic habitat, light habitat, and plant communities) (Fig. 1). The similarity is due to the fact that topography, light, and plant community co-vary at the study site because of their mutual dependence^18^. Thus, we defined the comprehensive habitat types jointly based on topography, light, and plant communities. Comprehensive habitat1 (n = 43) or comprehensive habitat2 (n = 87) were classified by PC1 (Fig. 1). The two comprehensive habitats were delineated based on the PCA axis of convex concave degree, leaf area index, average leaf angle, and species abundance of *Q. aliena* var. *acuteserrata* (Table S1). PC1 explained 72.6% of the total variation, largely reflecting variation in abundance of *Q. aliena* var. *acuteserrata* (Table S1).

**Figure 2:**
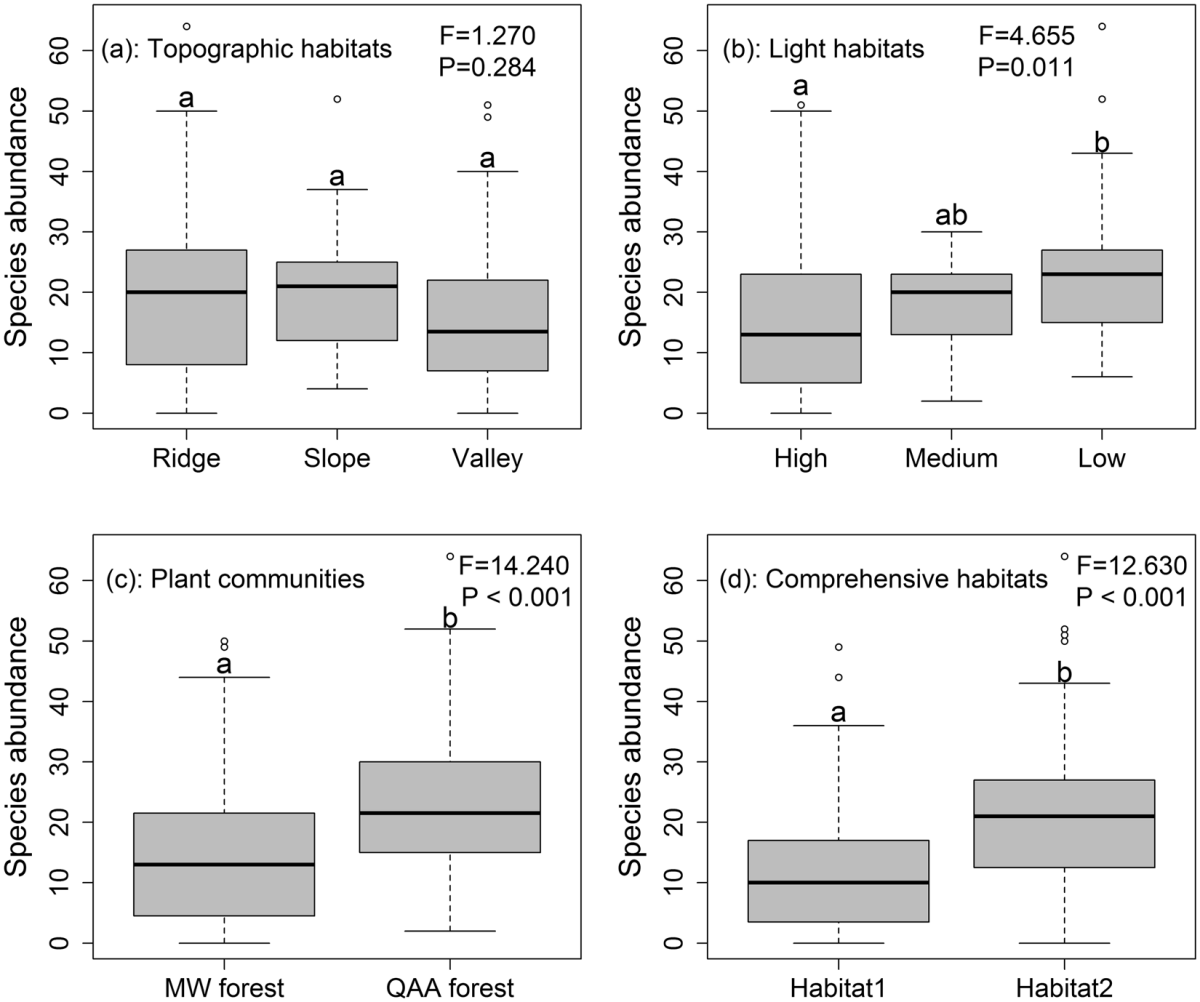
Species abundance of the sporocarps of macrofungi in topographic habitats (**a**), light habitats (**b**), plant communities (**c**), and comprehensive habitats (**d**) as demonstrated by boxplot with median and 95% confidence intervals displayed. Bars without shared letters indicate significant differences after adjustment by the Bonferroni method.

Of the 217 macrofungal species, 122 were recorded from the valley, 83 from the slope, and 181 from the ridge. A total of 159, 61, and 164 species were recorded from the high-light, medium-light, and low-light habitats, respectively. A total of 177 species were recorded from the MW forest, and 155 from the QAA forest. A total of 120 species were recorded from comprehensive habitat1, and 199 from comprehensive habitat2. Macrofungal species abundances and richness differed among high-light, medium-light, and low-light habitats; between MW and QAA forest; and comprehensive habitat1 and comprehensive habitat2, but not among valley, slope, or ridge microhabitats (Figs 2 and S2).

**Figure 3:**
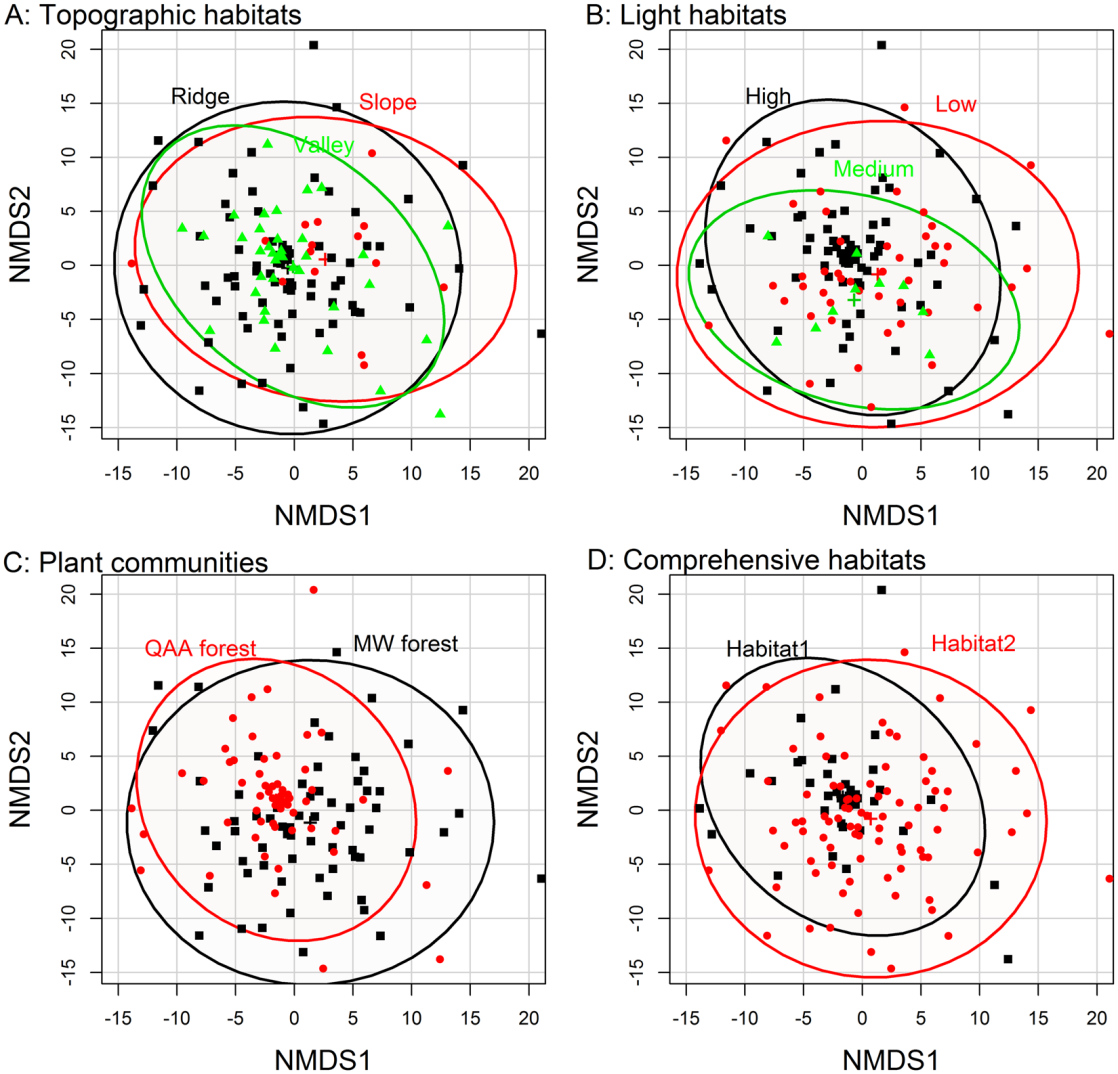
Nonmetric multidimensional scaling (NMDS) of the sporocarp composition of macrofungi. Points are color-coded according to habitat type, and 95% confidence ellipses are overlain.

**Figure 4:**
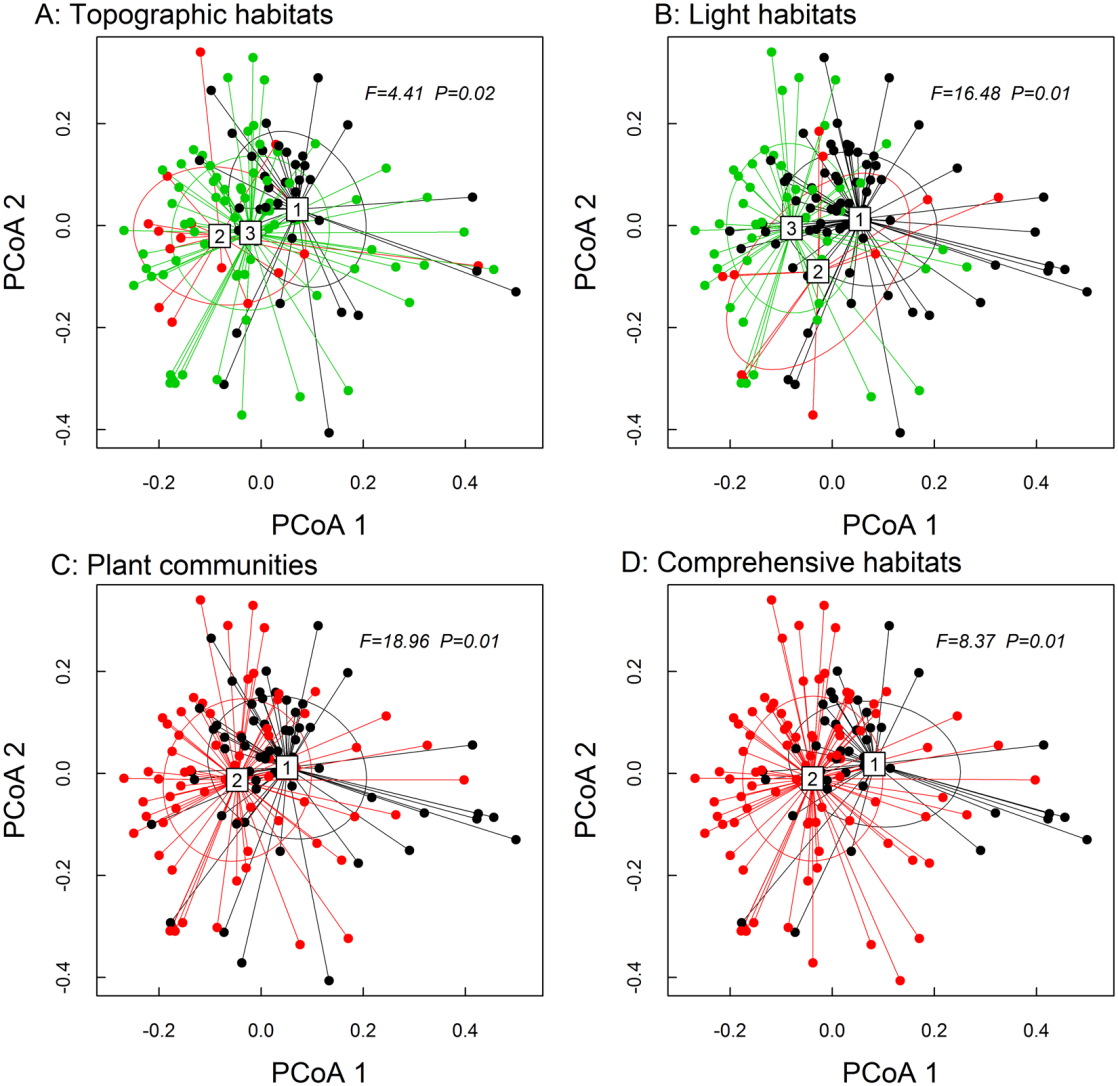
Effect of microhabitat types on beta diversity of the sporocarp community of macrofungi by running the betadisper function. ANOVA was applied to test these distances differed among the microhabitats. Points are color-coded according to habitat type, and 95% confidence ellipses are overlain. (**A**) ‘1’ is valley, ‘2’ is slope, and ‘3’ is ridge; (**B**) ‘1’ is high-light habitat, ‘2’ is medium-light habitat, and ‘3’ is low-light habitat; (**C**) ‘1’ is QAA forest, and ‘2’ is MW forest; (**D**) ‘1’ is comprehensive habitat 1, and ‘2’ is comprehensive habitat 2.

NMDS followed by PERMANOVA showed no significant differences in the sporocarp compositions of macrofungi among the topographic habitats (A: *DF* = 2, *F* = 1.054, *P* = 0.381). Significant differences were found in the sporocarp composition of macrofungi among the light habitats (B: *DF* = 2, *F* = 2.372, *P* = 0.044), plant communities (C: *DF* = 1, *F* = 6.651, *P* = 0.001), and the comprehensive habitat (D: *DF* = 1, *F* = 4.411, *P* = 0.014) (Fig. 3). Betadisper analysis followed by ANOVA test showed significant differences in the community dispersion of macrofungi among the topographic habitats (A: *DF* = 2, *F* = 4.405, *P* = 0.014), light habitats (B: *DF* = 2, *F* = 16.478, *P* < 0.001), plant communities (C: *DF* = 1, *F* = 18.958, *P* < 0.001), and the comprehensive habitat (D: *DF* = 1, *F* = 8.368, *P* = 0.005) (Fig. 4).

### Species–habitat associations

Indicator Species Analysis showed that the characteristics of the macrofungal assemblages varied among the habitats. For example, the macrofungal community in MW forest was mainly composed of *Russula vinosa*, *Russula decolorans*, and *Lactarius hatsudake*, among others. By contrast, *Daedalea dickinsii*, *Marasmius epiphyllus*, and *Collybia acervata* were dominant in QAA forest (Table S2).

**Figure 5:**
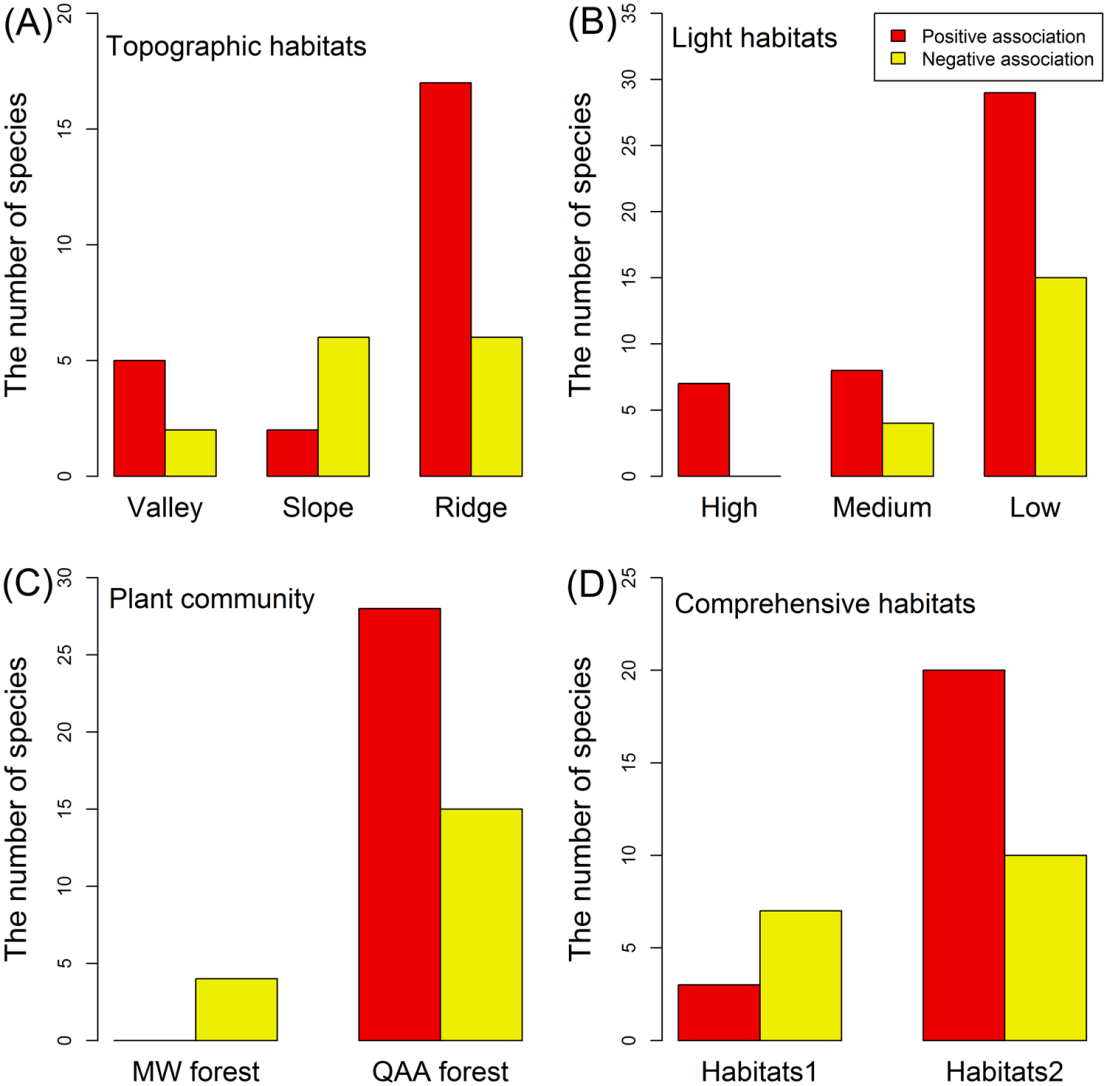
Number of species associated with topographic habitats (**A**), light habitats (**B**), plant communities (**C**), and comprehensive habitats (**D**). Torus-translation randomization tests were used to assess the significance of habitat associations for macrofungal species (see text for details).

Based on the Torus-translation tests, five, two, and seventeen species showed positive association (*p* < 0.050) to valley, slope, and ridge habitat, respectively. Twenty-nine positive associations were detected at the low-light habitat, compared to seven and eight at the high- and medium-light habitats, respectively. Twenty-eight positive associations were detected at the QAA forest, compared to zero at the MW forest. Twenty positive associations were detected for comprehensive habitats 2, compared to three for comprehensive habitats 1 (Fig. 5).

**Figure 6:**
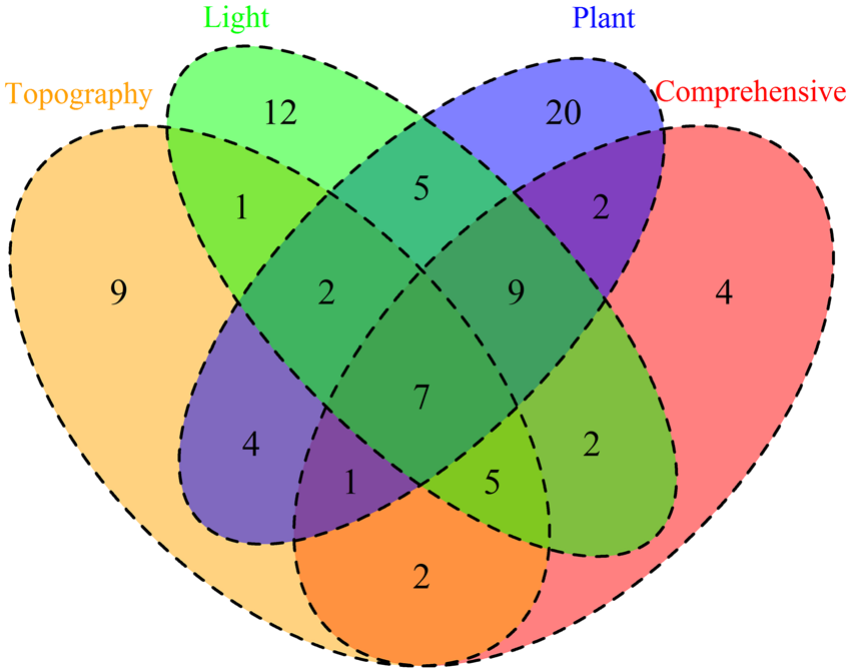
The number of macrofungal species associated with habitat types among topographic habitats, light habitats, plant communities, and comprehensive habitats.

A total of 85 species examined were associated (positively or negatively associated) with one or more of the habitat types (topographic habitat, light habitat, plant communities, or comprehensive habitats) (85/125, 68%). Compared with the light habitats and plant communities (40.0% and 34.4% positive associations, respectively), a lower ratio of species-habitat associations were detected (24.8%) with respect to topographic habitats. A total of 9, 12, and 20 species examined were associated only with one habitat type (topographic habitat, or light habitat, or plant community). Four species showed association only with the comprehensive habitats (Fig. 6). The results of microhabitat associations of the macrofungal species with topographic habitats, light habitats, plant communities, and comprehensive habitats are shown in Tables S3–S6, respectively.

### Sporocarp-environment relations among different microhabitats

Mantel tests showed that the factors related to the macrofungal species composition differed among the various microhabitats (Table S7). For example, macrofungal species composition were correlated to plant species composition in the comprehensive habitat1, but to average leaf angle, canopy cover, plant stand density, and plant basal area in comprehensive habitat2. Geographic distance was related to the macrofungal community in the valley, MW forest, and comprehensive habitat1 (Table S7).

**Figure 7:**
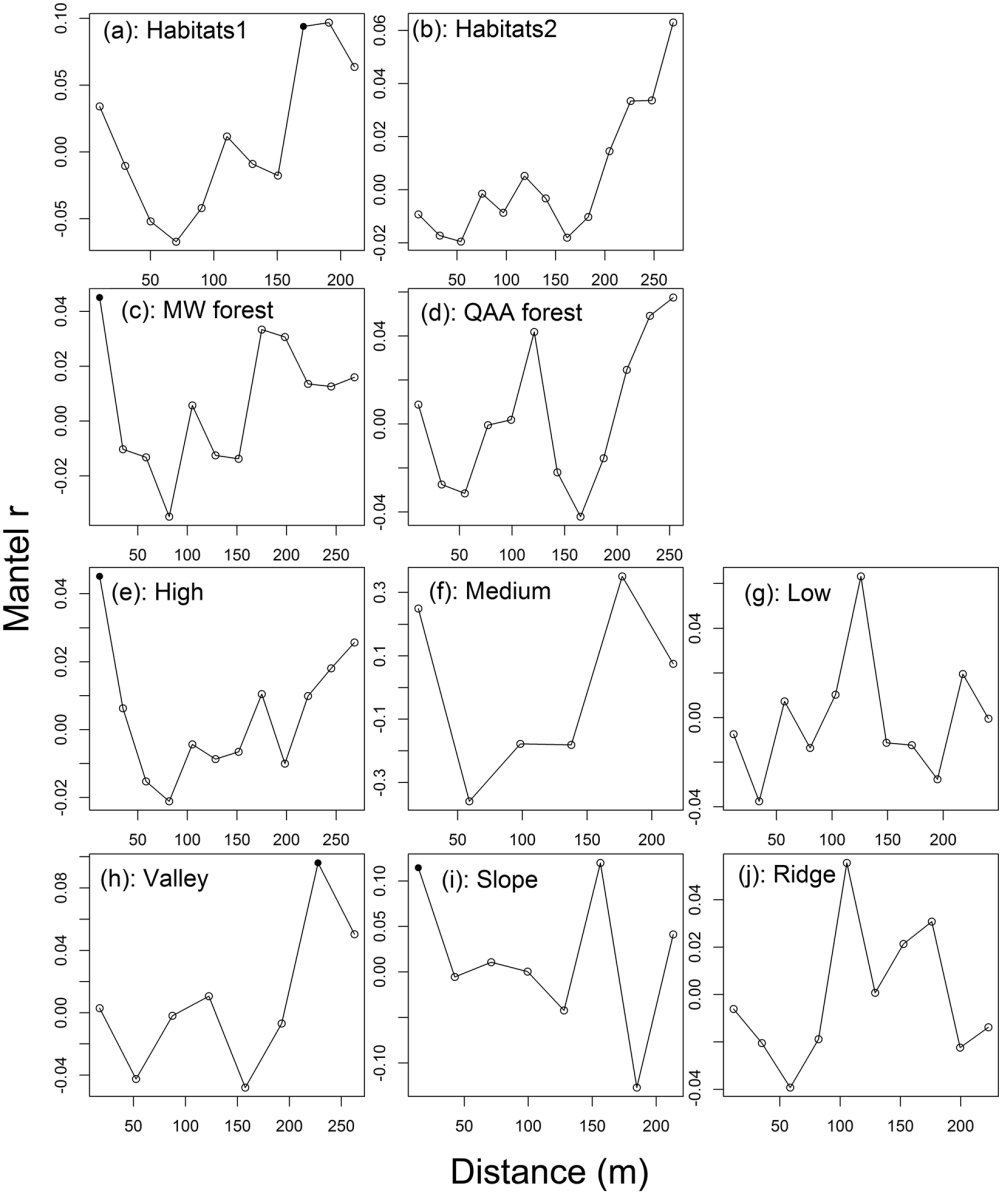
Mantel correlograms showing the correlation between partial residuals in sporocarp composition and spatial proximity for subplots within different distance classes in comprehensive habitats (**a** and **b**), plant communities (**b** and **c**), light habitats (**e**,**f**, and **g**), and topographic habitats (**h**,**i**, and **j**). Closed circles, P < 0.05; open circles, P > 0.05.

The final MRM models showed that different combinations of environmental variables were correlated with the sporocarp composition of the macrofungal species in the various microhabitats (Table 1). In addition, Mantel correlograms demonstrated significant spatial correlation between the macrofungal community and geographic distance in the following: at a spatial scale of 160 m in the comprehensive habitat1; 20 m in the MW forest and high-light habitat; and 240 m in the valley. However, no such correlation was found in the ridge, low-light habitat, QAA forest, and comprehensive habitat2 (Fig. 7).

**Table 1:** Multiple regressions on distance matrices (MRM) of sporocarp compositions as predicted by topographical, light, plant and spatial distance variables in the different microhabitats.

Habitats types	Distance matrix	Slope	*P*	Independent contribution (%)	Model parameters
**Topographic habitats**					
Valley	Spatial distance	<0.001	<0.001	2.50	*R*^2^ = 0.090, *p* = 0.040
	Plant species composition	0.072	0.020	6.48	
Slope	Plant richness	0.369	0.040		*R*^2^ = 0.154, *p* = 0.040
Ridge	Convex concave	<0.001	0.030	3.42	*R*^2^ = 0.144, *p* = 0.010
	Average leaf angle	0.093	0.050	1.90	
	Elevation	<0.001	0.010	2.90	
	Plant basal area	0.007	0.010	6.22	
**Light habitats**					
High	No variable retained in the final model				
Medium	Direct radiation	0.002	0.047	16.56	*R*^2^ = 0.288, *p* = 0.020
	Plant richness	0.242	0.050	12.19	
Low	Slope	0.149	0.020	8.23	*R*^2^ = 0.286, *p* = 0.030
	Aspect	0.121	0.030	7.22	
	Plant basal area	0.006	0.050	4.20	
	Canopy cover	<0.001	<0.001	2.60	
	Light transmittance	0.008	0.010	6.30	
**Plant community**					
MW forest	Slope	0.105	0.045	3.31	*R*^2^ = 0.099, *p* = 0.020
	Plant species composition	0.056	0.045	4.37	
	Spatial distance	0.005	0.040	2.21	
QAA forest	Elevation	<0.001	0.050	1.38	*R*^2^ = 0.109, *p* = 0.050
	Total radiation	0.002	0.040	3.21	
	Stand density	0.017	0.050	3.05	
	Plant basal area	0.005	0.040	3.28	
**Comprehensive habitats**					
Habitats1	Plant species composition	0.112	0.040	12.37	*R*^2^ = 0.216, *p* = 0.04
	Spatial distance	0.036	0.020	9.19	
Habitats2	Canopy cover	19.944	0.050	4.20	*R*^2^ = 0.147, *p* = 0.04
	Average leaf angle	0.117	0.040	3.80	
	Plant basal area	0.004	0.020	2.70	
	Stand density	0.012	0.045	4.01

## Discussion

In this study, our results showed different microhabitats favor occurrence of different macrofungal species, and sporocarps -environment relation varied among the different microhabitats at this temperate mountain forest locality. Together these findings suggest that that microhabitat specialization is important for macrofungal diversity at local scales.

### Different microhabitat preferences

In this study, we found that the characteristics of macrofungal sporocarp assemblages differed among the different microhabitats. A total of 85 species (68%) were associated with specific microhabitats (positively or negatively associated). This result may indicate microhabitat partitioning of the sporocarp community. In addition, fewer macrofungal species had specific preferences with respect to topographic microhabitats than with light microhabitats and plant communities. Hence, the effect of topographic microhabitat on the sporocarp community structure was not as important as expected. Woody plants constitute the structure of forest ecosystems, shaping factors such as light availability, humidity and litter composition^28–30^. In accordance with this perspective, more sporocarps of macrofungal species showed the strongest associations with light microhabitat and plant community in this study. Topography, light availability, and plant community are interrelated and interdependent in forest ecosystems^18^. However, our results showed that only nine species had simultaneous associations with topographic microhabitats, light microhabitats, and plant communities. Considering comprehensive microhabitats, 53 macrofungal species (53/85, 62%) still showed significant association with topographic microhabitats, light microhabitats, or plant communities, but no association was found with comprehensive microhabitats (Fig. 5).

In forested ecosystems, ridge and valley are the most common microhabitats associated with distinct species communities^20^. In general, valleys have more litter and soil nutrients than the ridge habitat^20,31^. However, our results showed that more macrofungal species displayed positive association with the ridge. This phenomenon may be due to the fact that most sporocarps of macrofungi are better at extracting nutrients from the soil^1^. Soil nutrients are generally not a limiting factor for sporocarp growth^32^. Several studies have also reported that good aeration is beneficial to the growth of sporocarp and spore dispersal of macrofungi^1,33,34^. Another reason is that the understory light availability is better on the ridge than in the valley (Fig. S3), and sporocarp of macrofungi may not prefer high-light habitats^35^. Thus, more macrofungal species were distributed in the ridge. In addition, the results indicated that more macrofungal species showed positive association with low-light habitat, but some macrofungi also showed negative association the low-light habitat. The growth of most sporocarps is related to light, and strong light may inhibit or even kill mycelia^1^. Strong light also facilitates water evaporation, and the humidity level is high under dense canopy cover^36^. Therefore, more macrofungal species showed positive association with low-light habitat. Although sporocarp of macrofungi does not favor strong light, suitable light can help some sporocarp grow^1,35^. Thus, a few macrofungal species showed negative association with low-light habitat. In addition, more macrofungal species showed positive than negative associations with the QAA forest, which may because the QAA is an ectomycorrhizal host tree, which must be affective to communities of macrofungi with mycorrhizal lifestyle. This may also reflect that the microhabitat under the canopy, such soil and light environment, created by QAA forest was suitable for the growth of many sporocarps of macrofungal species. Nouhra *et al*. also found that two different forest stands, each dominated by different species, exhibited different sporocarp communities^37^. Overall, these findings show that different sporocarps of macrofungal species have distinct specific microhabitat preferences in the temperate mountain forest.

### Sporocarp-environment relations depended on the different microhabitats

Climate variables such as temperature and precipitation are known to be major determinants for interannual variation or broad-scale patterns in sporocarp formation^38–40^. However, fine-scale processes influence the responsiveness of species to environment factors (such as topography, plant community, and light availability), often in complex ways^41^. The role of microhabitat partitioning processes for macrofungal species diversity has been poorly documented. Our results indicated that sporocarp-environment relations depend on the different microhabitats. For example, plant basal area was correlated with the sporocarp composition of the macrofungal species in the ridge, QAA forest, low-light habitat, and comprehensive habitat2, but not in the valley habitat, MW forest, high-light habitat, and comprehensive habitat1. At the study site, plant basal area was higher in ridge, QAA forest, low-light habitat, and comprehensive habitat2 than in the valley, MW forest, high-light habitat, and comprehensive habitat1 (Fig. S3). Basal area was related to the canopy, and larger basal area resulted in worse lighting conditions^42^. Several studies have demonstrated that strong light may inhibit or even kill some sporocarps of macrofungal species^1,5^, perhaps due to destruction of certain vitamins, essential for macrofungal growth^1^. In addition, composition of plant species was correlated with the sporocarp composition of the macrofungal species in the valley, MW forest, and comprehensive habitat1, but not in the ridge, QAA forest, and comprehensive habitat2. This phenomenon may be a result of the dominance of *Q. aliena* var. *acuteserrata* (accounting for 27.87% of total plant basal area), which was mainly distributed on the ridge, QAA forest, and comprehensive habitat2 (Fig. 1C). No dominant species were found in the valley, MW forest, and comprehensive habitat1.

Dispersal limitation has been reported to be one of important factors affecting macrofungal community in temperate deciduous broad-leaved forest^35^. In our study, geographic distance was correlated with these sporocarp compositions in the valley, MW forest, high-light habitat, and comprehensive habitat1, but not in the ridge habitat, QAA forest, low-light habitat, and comprehensive habitat2. This result may suggest that dispersal limitation is a stronger factor in the valley, MW forest, high-light habitat, and comprehensive habitat1 than in contrasting microhabitats^43^. One possible explanation is that the higher abundance and richness of sporocarp in the latter microhabitats (Figs 2, S1 and S2). Notably, the good aeration in the ridge microhabitat would promote growth of sporocarp and spore dispersal of macrofungi^1^. Further, the greater number of sporocarp of macrofungal species associated with QAA forest could reflect that *Q. aliena* var. *acuteserrata*, as the dominant species and with a denser canopy than mixed wood forest, created more continuous macrofungi-suitable low-light habitats^1^, reduing dispersal limitation.

## Conclusions

In conclusion, our study finds that many of the macrofungal species exhibit distinct microhabitat preferences. We furthermore find that sporocarp-environment relations differ among microhabitats defined by topography, understory light availability, and plant communities. Together these findings suggest that microhabitat specialization is important for sporocarp formation and potentially important for the maintenance of macrofungal species diversity. These findings underscore that in sustainable forest management, diverse microhabitats should be maintained, in terms of both vegetation structure and composition and abiotic conditions, since microhabitat variability will promote macrofungi diversity.

In this study, we only studied the effect of microhabitat specialization on the small-scale distribution macrofungi based on sporocarps. Temporal variation may affect these patterns, as sporocarp production is season-dependent and may also vary strongly among years. Furthermore, many fungal species may be present in a community at any given time without producing sporocarps. Therefore, future studies should assess the importance of the below-ground fungal community and temporal variation in sporocarp production for the patterns identified here.

## Material and Methods

### Study site and sampling

**Figure 8:**
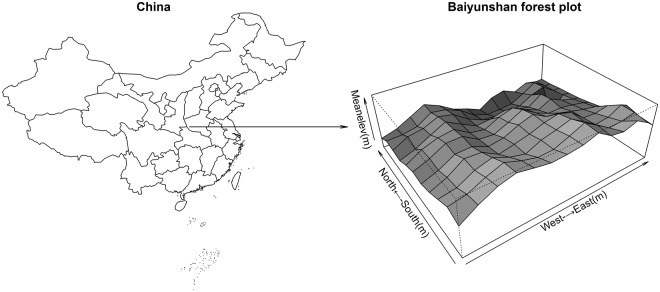
Topographic map of the 5-ha Baiyunshan permanent plot. The map was created using R3.4.2 software^43^ (https://www.r-project.org/).

The study was conducted in a 5-ha plot in a temperate deciduous broad-leaved forest in the Baiyunshan National Nature Reserve, East China (111°48′–112°16′ E, 33°33′–33°56′ N, *c*. 168 km^2^, 1538–1600 m above sea level) (Fig. 8). The study site has an annual mean temperature of 13.5 °C and annual mean precipitation of 1200 mm. The 5 ha plot contains secondary forest that was disturbed by charcoal production approximately 80 years ago. Most of the forest is currently at intermediate successional stages. All stems ≥1 cm diameters at breast height (DBH) in the Baiyunshan plot were tagged, mapped, and measured^44^.

The physiological characteristics of sporocarps of epigeous macrofungi differ from those of plants^1^. Spatial variations in the epigeous macrofungal community have not been well elucidated because of unsystematic and incomplete fungal monitoring efforts^45^. Forest dynamics plot is an integrated monitoring system that provides abundant environmental information for monitoring research of sporocarps of epigeous macrofungi^44^. In this study, macrofungal censuses were undertaken during the early rainy season from May to October in 2016. The surveys were conducted six times in late monthly. We collected the fruiting bodies of all epigeous macroscopic fungi, including soil macrofungi, rotten-wood macrofungi, litter macrofungi, and living-tree macrofungi, in the 5-ha plot with 130 subplots. There total amount of sporocarps per plot was used in the analysis. We carefully checked in the plot observable from every corner of 20 m × 20 m subplots to avoid overlooking any sporocarps of macrofungi. Macrofungal collectors have had professional training before macrofungal collection with regard to the basic characteristics of sporocarps and different methods of collection in the field. Sporocarps of macrofungi were dried and stored for identification, which was performed using macroscopic morphology and microscopic characteristics. A total of 2433 macrofungal fruiting bodies from 217 species were recorded (Tables S8).

Based on Harms *et al*.^9^ and Valencia *et al*.^46^, four topographic attributes, namely, elevation, convex concave, slope, and aspect, were calculated for each 20 m × 20 m subplot in the plot. A negative convex concave value indicated a hollow or local depression, while a positive convex concave value indicated a hillock in the plot. Aspect was represented by sin (aspect) and cos (aspect).

Light was surveyed by taking hemispherical photographs with an ultra-wide-angle fisheye lens (F2.8 EX DC, SIGMA, Japan) attached to a Canon 7D camera (EOS60D, Canon, Japan) body at the center of each 10 m × 10 m subplot in the permanent plot. The camera was leveled horizontally at 1.3 m above the ground and oriented to the true north using a compass with a spirit level^47^. We processed the images using the Gap Light Analyser software (version 2.0)^48^. Leaf area index, average leaf angle, canopy cover, total radiation, scattered radiation, direct radiation, and light transmittance were determined. The light environment in every 20 m × 20 m subplot was obtained by the mean value of four 10 m × 10 m subplots. Inaccurate readings caused by direct sunlight were avoided by surveying light early in the morning, late dusk, or on overcast days whenever possible. The light environment varied greatly from spring to late autumn in the deciduous broad-leaved forest. Thus, light were surveyed monthly from May to October in 2016. The mean of six surveys were used to represent the light environment of the forest community (Fig. S1).

In the 5-ha plot, 17, 963 individual woody plants (with DBH ≥1 cm) belonging to 55 families and 93 species were previously identified^23^. Of the 93 woody plant species, *Quercus aliena* var. *acuteserrata* is the dominant species (accounting for 27.87% of the total plant basal area). The 5-ha plot was divided into 130 subplots, in which various descriptors, i.e., plant stand density, plant basal area, and plant richness of the woody plant community, were measured. Plant stand density indicated the number of individual trees. Plant basal area was calculated as π × *R*^2^, where *R* is the radius at the height of 1.3 m. Plant richness was the number of species.

### Data analysis

Multivariate regression tree (MRT) analysis has been frequently performed to define microhabitat types, especially in studies based on the forest dynamics plot^8,9,16,20^. In this study, MRT was computed to delineate microhabitat types similar in environmental factors (topography, or light, or plant communities, or a combination of these factors) and sporocarp composition of macrofungal species. Three topographic habitats, three light habitats, two plant communities, and two comprehensive habitats were delineated by MRT. Multivariate regression tree analysis was conducted using the MVPART function in the mvpart package^49^. Comprehensive habitats were delineated based on the principal component analysis (PCA) of crucial environment factors in habitat classification of topography, light, and plant communities. PCA was performed using the vegan package^50^. One-way ANOVA was applied to test for differences in the abundance and richness of sporocarps of macrofungal species among these microhabitats.

The sporocarp composition of macrofungi was analyzed by ordination using nonmetric multidimensional scaling (NMDS) with the Bray-Curtis dissimilarity, and the habitat types were fitted as centroids onto the NMDS graph using the envfit function. Permutational multivariate ANOVA (PERMANOVA) was applied to explore the significant differences based on 999 permutations. NMDS was conducted using the metaMDS command in the vegan package (Oksanen *et al*.^50^).

Next, we assessed the impact of microhabitat types on beta diversity of the sporocarp community of macrofungi by running the betadisper function on the species matrix to calculate the distance from each microhabitat to treatment centroid in multivariate space. Once we obtained plot-centroid distances, we used an ANOVA to test whether these distances differed among microhabitats defined by topography, understory light availability, and plant communities. Betadisper test was conducted using the betadisper command in the vegan package^50^.

Indicator species analysis was computed to delineate the sporocarp composition of the macrofungal species among topographic habitats, light habitats, plant communities, and comprehensive habitats. The dependent variable was species abundance matrix of sporocarps in the Indicator species analysis. Indicator species analysis was performed using the indicspecies package^51^.

The habitat association method (Torus-translation test) proposed by Harms *et al*.^9^ allowed us to further assess the existence of microhabitat associations for macrofungi. This method is more conservative than goodness-of-fit tests or the randomized habitat method, because it largely accounts for spatial autocorrelation. The basic concept of the Torus-translation test is to calculate randomly the real distribution probability of a species in each habitat. Whether a species was significantly associated with a habitat could be determined using such probability. Further details on this method are given in Harms *et al*.^9^. In this paper, we defined habitats on the basis of a 20 m × 20 m grid of quadrants in the Baiyunshan permanent plot, and the results yielded one real and 519 Torus-translated habitat maps. We separately analyzed the topographic habitats, light habitats, plant communities, and comprehensive habitats and considered that *p* < 0.050 reflected spatial association (either for negative or positive values) between the analyzed species and the microhabitat. The dependent variable was species abundance matrix of sporocarps in the Torus-translation test. Habitat associations were only tested for species with more than five individuals in the entire 5-ha plot. Nearly 57.6% of the species (125/ 217) recorded greater than 1 individual per hectare in the entire 5-ha plot. For the Torus-translation test, we used the R code developed by Harms *et al*.^9^ and Comita *et al*.^8^.

For each microhabitat type, Bary-Curtis dissimilarities were calculated to construct distance matrices of macrofungal community, topographic attributes (slope, aspect, elevation, and convex concave), light attributes (leaf area index, average leaf angle, canopy cover, total radiation, scattered radiation, direct radiation, and light transmittance), plant community (stand density, basal area, species richness, and species composition), and geographic distance. Correlations among the distance matrices listed above were explored by Mantel tests based on Pearson method and 99 permutations. Mantel test is a correlation between entries of two dissimilarity matrices and performed in the ecodist package^52^.

The extent to which the sporocarp community was influenced by these factors was quantitatively tested by constructing MRM models to include distance matrices significantly related to the sporocarp community according to the results of Mantel tests. Then, the MRM models were simplified until *p* < 0.050 for all distance matrices by stepwise backward selection. Compared to forward selection, the advantage of backward selection is that it allows some variables of low contribution value to enter into the model, and can avoid by one or two dominant variables of interference^53^. If more than one environmental factor was retained in the final MRM model, the independent contribution of each environmental factor was explored by hierarchical partitioning. Hierarchical partitioning was conducted in the hier.part package^54^. The partial residuals in the sporocarp community were calculated by partialling out the effects of significant environmental factors retained in the final MRM model. The geographic distances between pairs of subplots (ranging from 20 m to 300 m) were calculated based on the center position of each 20 m × 20 m subplot. The correlation between the partial residuals in the sporocarp community and geographic distance at different spatial scales were tested by Mantel correlograms. Mantel correlograms, and MRM were performed in the ecodist package^52^.

All statistical analyses were conducted in R3.4.0 (R Development Core Team, http://www.Rproject.org)^55^.

## Electronic supplementary material

Supplementary information

## Data Availability

Te dataset analyzed during the current study is available from corresponding author on reasonable request.
