# Supplementary material for: Macrofungal species distributions depend on habitat partitioning of topography, light, and vegetation in a temperate mountain forest
Source: Sci Rep. 2018 Sep 11;8:13589. doi: 10.1038/s41598-018-31795-7 (PMC6134103; doi:10.1038/s41598-018-31795-7)
Supplement: Supplementary file 1 — Supplementary information [file 41598_2018_31795_MOESM1_ESM.pdf]

Macrofungal species distributions depend on habitat partitioning of topography, light, and vegetation in a temperate mountain forest

Yun Chen[1,2,3]*, Zhiliang Yuan[1]*, Shuai Bi[1], Xueying Wang[1], Yongzhong Ye[1,&], and Jens-Christian Svenning[2,3]

[1] College of Life Sciences, Henan Agricultural University, No.63 Agricultural Road, Zhengzhou 450002, China. [2]Section for Ecoinformatics and Biodiversity, Department of Bioscience, Aarhus University, Aarhus, Denmark. [3]Center for Biodiversity Dynamics in a Changing World (BIOCHANGE), Aarhus University, Aarhus, Denmark.

*These authors contributed equally to this work.

[&]Correspondence and requests for materials should be addressed to *Yongzhong Ye*. (email: yeyzh@163.com)

,

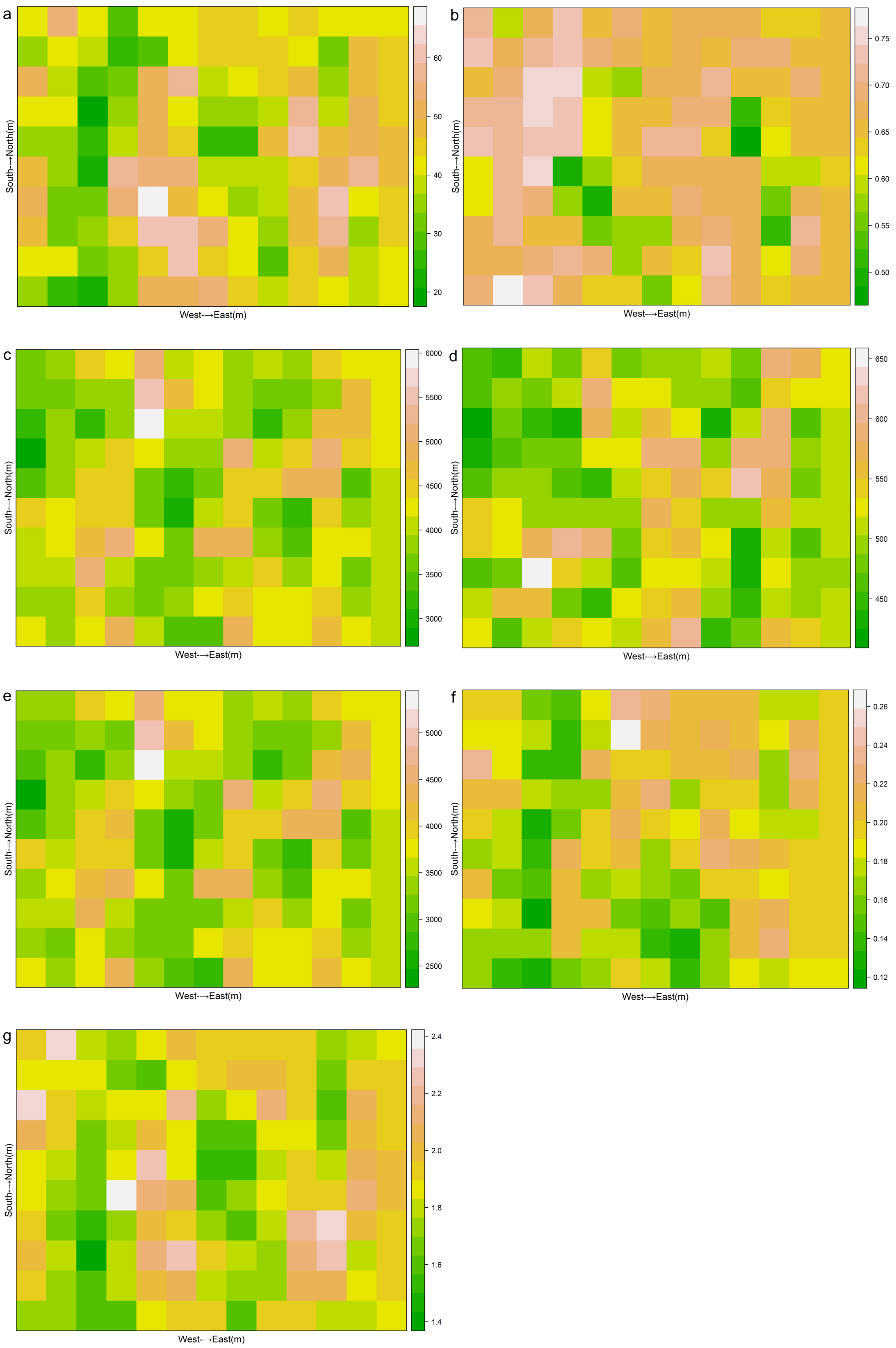

Fig. S1 Maps of the average leaf angle (a), canopy cover (b), total radiation (c), scattered radiation (d), direct radiation (e), light transmittance (f), leaf area index (g) in the 5-ha Baiyunshan permanent plot.

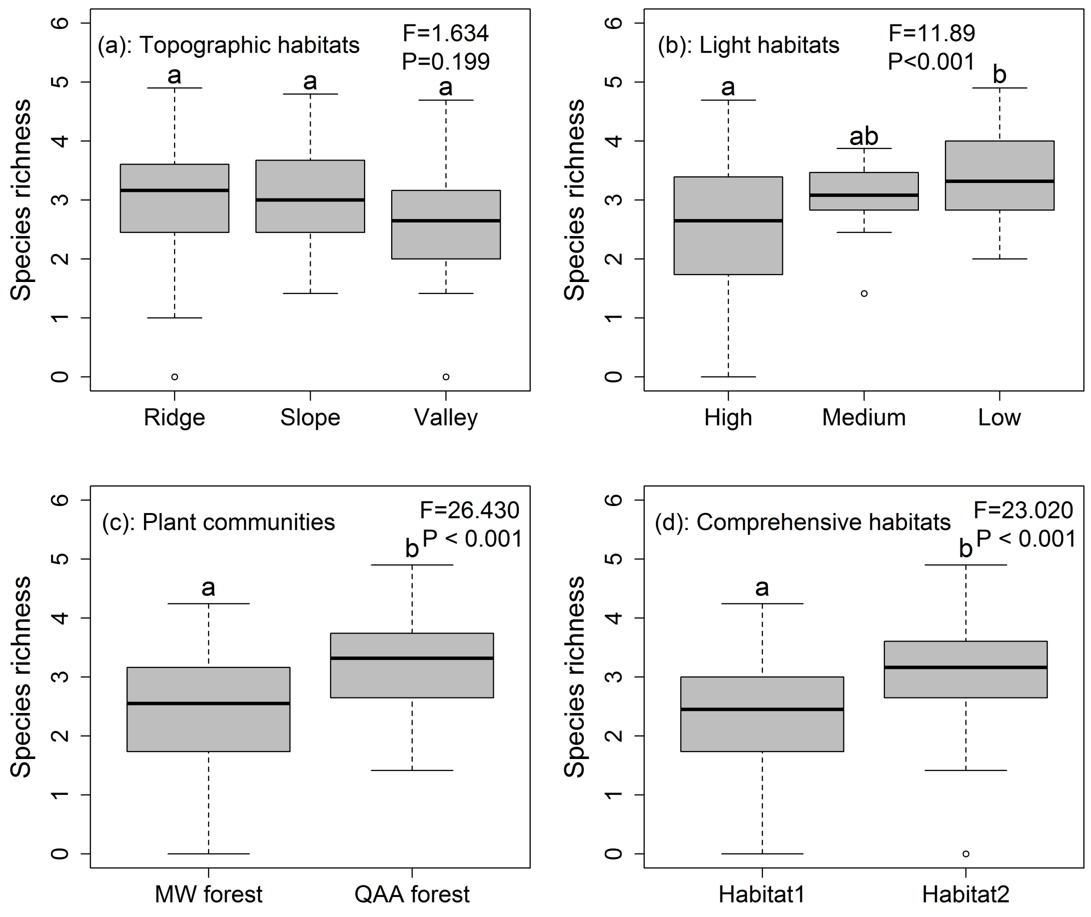

Fig. S2 Macrofungal species richness in topographic habitats (a), light habitats (b), plant communities (c), and comprehensive habitats (d) as demonstrated by boxplot with median and 95% confidence intervals displayed. Bars without shared letters indicate significant differences after adjustment by the Bonferroni method.

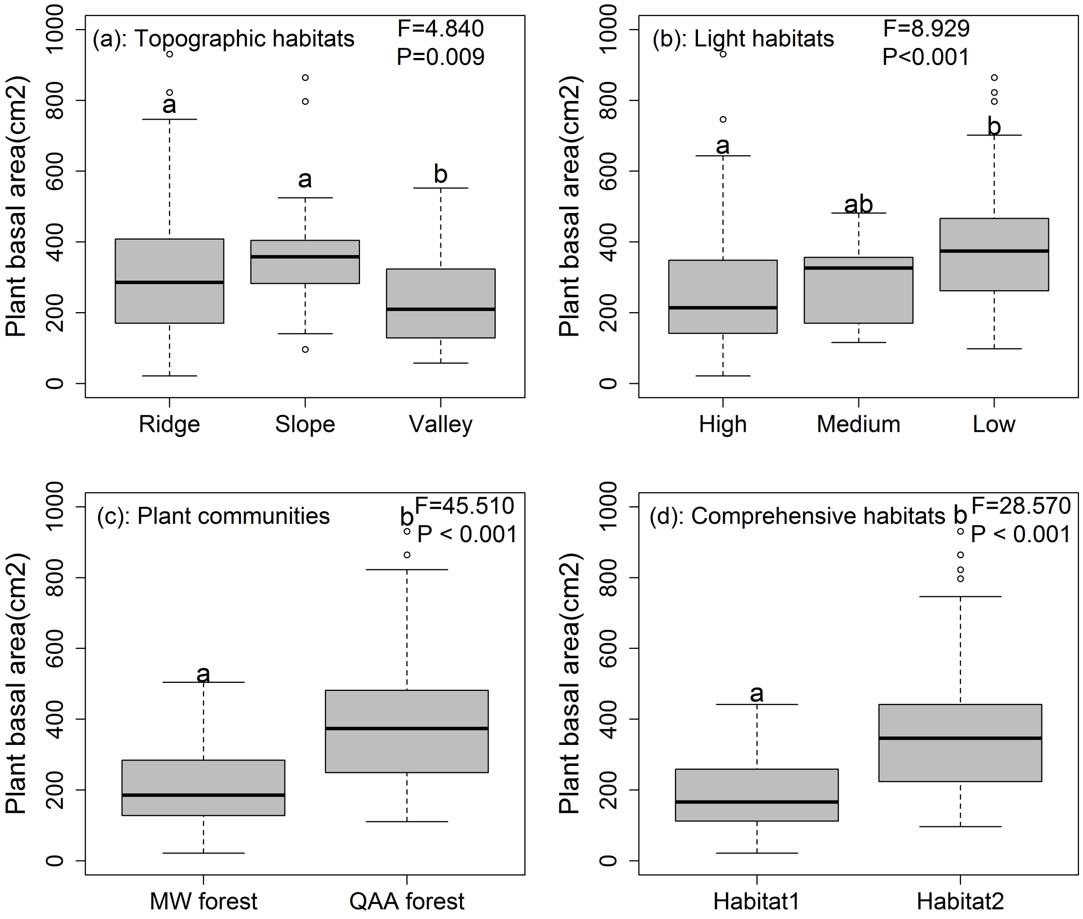

Fig. S3 Plant basal area in topographic habitats (a), light habitats (b), plant communities (c), and comprehensive habitats (d) as demonstrated by boxplot with median and 95% confidence intervals displayed. Bars without shared letters indicate significant differences after adjustment by the Bonferroni method.

Table S1 Principal component analysis (PCA) of leaf area index, average leaf angle, convex concave, and species abundance of QAA. QAA is *Quercus aliena* var. *acuteserrata*.

Factors	PC1	PC2	PC3	PC4
Leaf area index	0.049	-0.007	-0.070	<0.001
Average leaf angle (°)	3.651	-0.835	-4.199	<0.001
Convex concave (°)	1.104	8.597	-0.357	<0.001
Species abundance of QAA	15.286	0.421	-1.027	<0.001
Eigenvalue	657.534	198.090	49.672	0.013
Proportion explained	0.726	0.219	0.055	<0.001
Accumulative proportion	0.726	0.945	0.999	1.000

Table S2 Indicator species analysis for macrofungal community among topographic habitats, light habitats, plant communities, and comprehensive habitats, respectively.

Species	s.1	s.2	s.3	Index	Stat	*P* value
**Topographic habitats**	Ridge	Slope	Valley			
Russula vinosa	0	1	0	2	0.657	0.001
Merulius tremellosus	0	1	0	2	0.447	0.003
Marasmius epiphyllus	0	0	1	3	0.436	0.008
Psathyrella candolleana	0	1	0	2	0.467	0.008
Tylopilus plumbeoviolaceus	0	1	0	2	0.365	0.021
Russula decolorans	1	0	0	1	0.465	0.023
Lactarius piperatus	0	1	0	2	0.404	0.024
Oudemansiella mucida	0	0	1	3	0.351	0.024
Suillus luteus	0	1	0	2	0.386	0.026
Russula ballouii	0	1	0	2	0.325	0.036
Boletus pseudocalopus	0	1	0	2	0.384	0.037
Lactarius hatsudake	0	1	0	2	0.457	0.049
Leucocoprinus birnbaumii	0	0	1	3	0.281	0.049
**Light habitats**	High	Low	Medium			
Lactarius piperatus	0	0	1	3	0.514	0.003
Russula vesca	0	0	1	3	0.569	0.004
Inocybe asterospora	0	0	1	3	0.464	0.011
Trametes gibbosa	0	0	1	3	0.42	0.015
Tylopilus felleus	0	1	0	2	0.395	0.033
Russula vinosa	0	1	0	2	0.547	0.04
Calocera cornea	1	0	0	1	0.452	0.043
Russula albida	0	0	1	3	0.564	0.047
Russula lutea	0	1	0	2	0.488	0.05
**Plant communities**	MW forest	QAA forest				
Russula vinosa	1	0		1	0.603	0.002
Lactarius hatsudake	1	0		1	0.496	0.003
Russula decolorans	1	0		1	0.462	0.003
Boletus magnificus	1	0		1	0.377	0.004
Lactarius pallidus	1	0		1	0.436	0.012
Russula lutea	1	0		1	0.491	0.012
Tylopilus virens	1	0		1	0.331	0.02
Russula albida	1	0		1	0.571	0.021
Abortiporus biennis	1	0		1	0.383	0.023
Coriolus versicolor	1	0		1	0.423	0.024
Collybia acervata	0	1		2	0.28	0.025
Boletus pseudocalopus	1	0		1	0.343	0.028
Daedalea dickinsii	0	1		2	0.328	0.028
Lactarius camphoratus	1	0		1	0.302	0.03

Russula delica	1	0	1	0.383	0.03
Lactarius deliciosus	1	0	1	0.302	0.033
Xerocomus badius	1	0	1	0.302	0.038
Tylopilus felleus	1	0	1	0.326	0.039
Russula heterophylla	1	0	1	0.325	0.044
Volvariella volvacea	1	0	1	0.378	0.044
Marasmius epiphyllus	0	1	2	0.321	0.047
**Comprehensive habitats**	Habitat1	Habitat2			
Russula vinosa	0	1	2	0.606	0.002
Lactarius hatsudake	0	1	2	0.531	0.003
Russula delica	0	1	2	0.4	0.009
Russula decolorans	0	1	2	0.455	0.016
Russula albida	0	1	2	0.587	0.017
Boletus magnificus	0	1	2	0.356	0.027
Russula heterophylla	0	1	2	0.339	0.029
Tylopilus felleus	0	1	2	0.339	0.038
Marasmius epiphyllus	1	0	1	0.33	0.047

Table S3 Significant habitat associations of macrofungi species with topographic habitats (p = 0.05 level of significance for Torus-translations test). + : significant positive association; - : significant negative association; N: neutral association.

Species	Valley	Slope	Ridge
Amanita junquillea	N	N	+
Auriscalpium vulgare	N	N	+
Boletus pseudocalopus	+	N	N
Calocera cornea	N	-	+
Cantharellus infundibuliformis	N	N	+
Collybia dryophlia	N	N	+
Cortinarius melanotus	N	N	-
Cyclomyces fuscus	N	-	N
Hygrocybe miniata	N	N	+
Hypocrea argillacea	N	N	+
Inocybe asterospora	N	-	+
Laetiporus sulphureus	N	N	+
Marasmius epiphyllus	N	-	+
Marasmius neosessilis	N	N	+
Marasmius oreades	N	N	+
Naematoloma sublateritium	-	+	N
Oudemansiella mucida	N	-	+
Psathyrella candolleana	N	-	N
Psathyrella velutina	N	N	+
Russula ballouii	+	N	N
Russula decolorans	N	+	-
Russula heterophylla	N	N	-
Russula vesca	N	N	-
Russula vinosa	+	N	-
Sirobasidium magnum	N	N	+
Strobilurus stephanocystis	N	N	+
Suillus flavus	-	N	N
Suillus flavidus	+	N	N
Tricholoma terreum	+	N	N
Tylopilus felleus	N	N	-
Volvariella bombycina	N	N	+

Table S4 Significant habitat associations of macrofungi species with light habitats (p = 0.05 level of significance for Torus-translations test). + : significant positive association; - : significant negative association; N: neutral association.

Species	High	Medium	Low
Aleurodiscus amorphus	N	N	+
Amanita junquillea	N	N	+
Asterophora lycoperdoides	N	N	+
Bolbitius vitellinus	N	N	+
Boletus impolitus	+	N	N
Boletus zelleri	N	N	−
Calocera cornea	N	−	+
Cantharellus cibarius	N	+	−
Clitopilus prunulus	N	N	+
Collybia acervata	N	N	+
Daedalea dickinsii	N	N	+
Ditiola radicata	N	N	+
Favolus arcularius	N	N	+
Tricholoma terreum	N	+	N
Fomitopsis vinosa	N	N	+
Gerronema fibula	+	N	N
Gloeophyllum sepiarium	N	N	+
Hygrocybe miniata	N	N	+
Hygrophorus lucorum	+	N	N
Hypholoma appendiculatum	N	N	+
Hypocrea argillacea	N	N	+
Inocybe asterospora	+	−	N
Lactarius hatsudake	N	N	−
Lactarius piperatus	N	N	−
Marasmius epiphyllus	N	−	+
Marasmius neosessilis	N	N	+
Melanoleuca cognata	N	N	+
Oligoporus caesius	N	N	−
Oligoporus tephroleucus	N	N	+
Pluteus cervinus	N	N	+
Plutteus atricapillus	N	N	+
Psathyrella candolleana	N	N	+
Rhodophyllus quadratus	N	N	+
Russula albida	+	N	−
Russula flavida	N	+	−
Russula foetens	N	N	−
Russula heterophylla	N	+	−
Russula rosacea	N	N	+
Russula vesca	+	N	N
Russula vinosa	N	+	−

Scutellinia scutellata	N	−	+
Serpula lacrymana	N	N	+
Sirobasidium magnum	N	N	+
Suillus luteus	N	N	−
Termitomyces albiceps	N	N	+
Trametes gibbosa	+	N	−
Tylopilus felleus	N	+	−
Tylopilus virens	N	+	−
Xerocomus badius	N	+	−
Xeromphalina tenuipes	N	N	+

Table S5 Significant habitat associations of macrofungi species with plant community habitats (p = 0.05 level of significance for Torus-translations test). + : significant positive association; - : significant negative association; N: neutral association.

Species	Miscellaneous wood forest	Quercus aliena var. acuteserrata forest
Abortiporus biennis	N	−
Albatrellus dispansus	N	−
Amanita junquillea	N	+
Armillariella mellea	N	+
Asterophora lycoperdoides	N	+
Bolbitius vitellinus	N	+
Boletus luridus	N	+
Boletus magnificus	N	−
Boletus pseudocalopus	N	−
Calocera cornea	−	+
Cantharellus cibarius	N	−
Cantharellus lateritius	N	+
Clitopilus prunulus	N	+
Collybia acervata	−	+
Coriolus versicolor	N	−
Daedalea dickinsii	−	+
Ditiola radicata	N	+
Favolus arcularius	N	+
Gerronema fibula	N	+
Hirschioporus lacteus	N	+
Hygrocybe miniata	N	+
Hygrophorus ceraceus	N	+
Hypocrea argillacea	N	+
Laccaria proxima	N	+
Lactarius camphoratus	N	−
Lactarius deliciosus	N	−
Lactarius hatsudake	N	−
Marasmius epiphyllus	−	+
Marasmius neosessilis	N	+
Marasmius oreades	N	+
Oudemansiella mucida	N	+
Pluteus cervinus	N	+
Psathyrella velutina	N	+
Russula decolorans	N	−
Russula lutea	N	−
Russula vinosa	N	−
Serpula lacrymana	N	+
Sirobasidium magnum	N	+
Stereum gausapatum	N	+
Tylopilus felleus	N	−

Tylopilus virens	N	－
Volvariella bombycina	N	＋
Xerocomus badius	N	－

Table S6 Significant habitat associations of macrofungi species with comprehensive habitats (p = 0.05 level of significance for Torus-translations test). + : significant positive association; - : significant negative association; N: neutral association.

Species	Habitats1	Habitats2
Amanita junquillea	N	+
Oudemansiella mucida	N	+
Russula delica	N	-
Sirobasidium magnum	N	+
Collybia acervata	-	+
Serpula lacrymana	N	+
Hygrocybe punicea	N	+
Scutellinia scutellata	N	+
Lactarius hatsudake	+	-
Boletus magnificus	-	N
Pluteus cervinus	+	-
Calocera cornea	N	+
Tylopilus felleus	N	-
Favolus arcularius	N	+
Clitopilus prunulus	N	+
Tylopilus virens	-	N
Ditiola radicata	N	+
Daedalea dickinsii	N	+
Strobilurus stephanocystis	N	+
Hypocrea argillacea	N	+
Melanoleuca cognata	N	+
Russula decolorans	N	-
Russula heterophylla	N	-
Marasmius epiphyllus	-	+
Volvariella bombycina	N	+
Marasmius oreades	-	+
Coriolus versicolor	N	-
Psathyrella velutina	-	+
Coprinus plicatilis	-	+
Russula vinosa	+	-
Laccaria amethystea	N	-
Cortinarius melanotus	N	-

Table S7 Mantel tests of the relationships between macrofungal community composition and environment variables in topographic habitats, light habitats, plant community, and comprehensive habitats.

Distance matrix	Topographic habitats						Light habitats						Plant community				Comprehensive habitats			
	Valley		Slope		Ridge		High		Medium		Low		MW forest		QAA forest		Habitat1		Habitat2	
	R	*P*	*R*	*P*	*R*	*P*	*R*	*P*	*R*	*P*	*R*	*P*	*R*	*P*	*R*	*P*	*R*	*P*	*R*	*P*
Slope (°)	-0.111	0.826	-0.121	0.789	0.096	0.137	-0.100	0.906	0.066	0.312	0.286	0.032	0.195	0.048	-0.087	0.845	0.021	0.385	0.124	0.088
Aspect	-0.101	0.829	-0.121	0.791	0.096	0.142	-0.100	0.915	0.066	0.283	0.258	0.028	0.127	0.051	-0.087	0.845	0.022	0.414	0.124	0.079
Elevation (m)	-0.068	0.860	0.022	0.444	0.131	0.047	-0.087	0.942	-0.217	0.859	-0.042	0.638	-0.008	0.522	0.157	0.047	-0.058	0.722	-0.063	0.861
Convex concave (°)	-0.013	0.500	0.161	0.207	0.184	0.037	-0.072	0.745	-0.037	0.540	-0.154	0.906	-0.094	0.797	-0.121	0.870	-0.144	0.849	-0.100	0.860
Leaf area index	0.124	0.084	0.019	0.377	-0.014	0.571	0.127	0.076	0.326	0.202	-0.056	0.704	0.009	0.430	0.105	0.137	0.150	0.106	0.011	0.405
Average leaf angle (°)	0.109	0.109	0.096	0.296	0.137	0.047	-0.002	0.473	-0.097	0.484	0.126	0.109	0.044	0.145	0.046	0.271	-0.023	0.579	0.176	0.008
Canopy cover	-0.015	0.503	0.276	0.084	0.128	0.113	0.047	0.249	0.006	0.354	0.150	0.041	0.132	0.086	0.117	0.125	0.068	0.227	0.179	0.030
Total radiation (mol/(m^2-d))	0.091	0.194	0.146	0.230	-0.020	0.560	0.074	0.176	0.364	0.052	-0.063	0.688	-0.134	0.962	0.179	0.050	-0.018	0.541	0.009	0.417
Scattered radiation (mol/(m^2-d))	0.041	0.344	0.084	0.358	0.037	0.321	0.079	0.169	-0.144	0.792	0.041	0.307	-0.007	0.510	0.066	0.238	0.029	0.361	0.015	0.390
Direct radiation (mol/(m^2-d))	0.093	0.188	0.163	0.226	-0.023	0.592	0.074	0.213	0.407	0.028	-0.083	0.798	-0.148	0.982	0.125	0.159	0.017	-0.531	0.001	0.470
Light transmittance	-0.181	0.956	-0.061	0.623	0.118	0.066	-0.001	0.504	-0.010	0.502	0.312	0.018	0.002	0.442	-0.014	0.541	0.006	0.447	0.044	0.242
Plant species composition	0.255	0.004	0.099	0.345	0.135	0.153	0.139	0.061	-0.513	0.985	-0.135	0.823	0.221	0.043	-0.026	0.602	0.303	0.007	-0.044	0.643
Plant stand density	-0.034	0.590	-0.014	0.404	-0.031	0.640	0.076	0.162	-0.247	0.933	0.021	0.324	0.008	0.372	0.174	0.031	0.106	0.221	0.224	0.003
Plant basal area (cm^2)	-0.161	0.951	-0.203	0.799	0.249	0.006	0.048	0.269	-0.300	0.947	0.205	0.035	0.071	0.280	0.181	0.028	0.032	0.337	0.164	0.022
Plant richness	-0.054	0.602	0.391	0.047	-0.085	0.899	0.044	0.264	0.302	0.040	-0.108	0.869	-0.087	0.872	0.019	0.336	0.054	0.257	-0.098	0.941
Spatial distance	0.134	0.044	0.106	0.235	-0.063	0.891	0.121	0.092	-0.116	0.687	-0.018	0.579	0.182	0.042	-0.135	0.577	0.282	0.042	-0.046	0.834

Table S8 Macrofungal species and substrate types were recorded in Baiyunshan plot.

Species	Soil	Rotten-wood	Litter	Living tree
Agaricus abruptibulbus	0	12	0	4
Agaricus comtulus	2	0	0	0
Agaricus placomyces	3	0	0	0
Agaricus silvaticus	3	0	0	0
Agaricus silvicola	8	0	0	0
Albatrellus dispansus	2	0	0	0
Aleurodiscus amorphus	10	0	0	0
Amanita caesarea	0	4	0	0
Amanita ceciliae	6	0	0	0
Amanita citrina	1	0	2	0
Amanita excelsa	1	0	0	0
Amanita fulva	4	0	0	0
Amanita pantherina	2	0	0	0
Amanita parvipantherina	0	0	2	0
Amanita sculpta	4	0	0	0
Amanita spissa	2	0	0	0
Amanita spissacea	4	0	1	0
Amanita spreta	1	0	0	0
Amanita subjunquillea	1	0	0	0
Amanita verna	2	0	0	0
Amanita virosa	2	0	0	0
Armillaria luteovirens	2	0	0	0
Armillariella mellea	1	0	0	0
Asterophora lycoperdoides	9	0	0	0
Auriscalpium vulgare	1	0	0	0
Bjerkandera fumosa	13	0	0	0
Bolbitius vitellinus	4	0	5	0
Boletellus longicollis	10	0	0	0
Boletus albus	0	2	0	0
Boletus edulis	0	10	0	0
Boletus flammans	8	0	0	0
Boletus impolitus	0	0	0	4
Boletus luridus	5	0	0	0
Boletus magnificus	10	0	0	0
Boletus ornatipes	18	0	0	0
Boletus pseudocalopus	15	0	0	0
Boletus pulverulentus	16	0	0	0
Boletus retipes	1	0	0	0
Boletus speciosus	15	0	0	0
Boletus zelleri	6	0	0	0

Cantharellus cibarius	1	0	0	0
Cantharellus infundibuliformis	1	0	0	0
Cantharellus lateritius	7	0	0	0
Clitocybe odora	0	32	0	0
Clitopilus prunulus	6	0	0	0
Collybia acervata	9	0	0	0
Collybia dryophlia	12	0	0	0
Conocybe lactea	4	0	0	0
Coprinus plicatilis	3	0	0	0
Coriolus consors	1	0	0	0
Coriolus unicolor	4	0	0	0
Coriolus versicolor	0	6	0	0
Cortinarius crocolitus	1	0	0	0
Cortinarius largus	11	0	47	0
Crepidotus mollis	2	0	0	0
Cryptoporus volvatus	5	0	0	0
Cyclomyces fuscus	3	0	0	0
Cyptotrama chrysopeplum	0	2	0	0
Cystoderma granulosum	0	2	0	0
Dacrymyces palmatus	34	11	0	1
Daedalea biennis	10	0	0	0
Daedalea dickinsii	27	0	0	0
Dictyopanus pusillus	0	2	0	0
Favolus alveolaris	0	5	0	1
Favolus arcularius	0	6	0	0
Favolus mollis	2	0	0	0
Flammulina velutiper	0	14	0	0
Fomitopsis pinicola	0	2	0	0
Fomitopsis vinosa	0	5	0	0
Geastrum saccatum	0	3	0	0
Geastrum velutinum	0	4	0	0
Gerronema fibula	7	1	0	0
Gloeophyllum sepiarium	0	1	0	0
Gomphidius roseus	0	41	0	0
Grifola fronaosa	0	6	0	0
Gyrophana lacrymans	3	0	0	0
Gyroporus purpurinus	34	0	0	0
Hirschioporus lacteus	0	14	0	0
Hydnum repandum	14	0	0	0
Hygrocybe cantharellus	0	1	0	0
Hygrocybe punicea	10	0	0	0
Hygrophorus ceraceus	0	2	0	2

Hygrophorus lucorum	0	1	0	0
Hygrophorus miniatus	0	0	2	0
Hypholoma cinnabarinum	12	0	1	0
Hypocrea argillacea	2	4	0	0
Inocybe asterospora	0	0	4	0
Inocybe umbrinella	5	0	0	0
Inonotus sinensis	73	0	0	0
Ischnoderma resinosum	0	5	0	0
Laccaria amethystea	19	0	0	0
Laccaria laccata	2	0	0	0
Laccaria proxima	0	0	0	2
Laccaria vinaceoavellanea	0	10	0	1
Lactarius akahatus	25	0	0	0
Lactarius camphoratus	8	1	0	0
Lactarius controversus	4	0	0	0
Lactarius deliciosus	23	1	0	0
Lactarius fuliginosus	1	0	0	0
Lactarius gerardii	21	0	0	0
Lactarius glyciosmus	12	0	0	0
Lactarius hatsudake	24	0	0	0
Lactarius pallidus	3	0	0	0
Lactarius piperatus	3	0	0	0
Lactarius sanguifluus	3	0	0	0
Laetiporus sulphureus	43	0	0	0
Leccinum griseum	38	0	0	0
Leccinum scabrum	14	0	0	0
Lentinu edodes	8	0	0	0
Lenzites betulina	0	42	0	2
Lepista nuda	2	0	0	0
Lepista personata	1	0	0	0
Leucocoprinus birnbaumii	1	0	0	0
Limacella glioderma	0	1	0	0
Lycoperdon perlatum	1	0	0	0
Marasmius cohaerens	0	0	1	0
Marasmius epiphyllus	3	0	0	0
Marasmius maximus	3	0	0	0
Marasmius neosessilis	0	0	0	2
Marasmius oreades	10	1	1	0
Marasmius siccus	0	0	44	0
Melanoleuca cognata	5	0	0	0
Melanoleuca exscissa	0	4	0	0
Melanoleuca strictipes	24	5	6	9

Merulius tremellosus	0	0	1	0
Mycena citrinella	26	0	0	0
Mycena galericulata	9	0	0	0
Mycena lactea	2	0	0	0
Mycoleptodonoi des aitchisonii	16	0	0	0
Naematoloma fasciculare	0	3	0	0
Naematoloma sublateritium	0	0	1	0
Odontia queletii	1	0	2	0
Oligoporus caesius	3	0	0	0
Oligoporus tephroleucus	0	0	0	1
Oudemansiella mucida	0	1	0	0
Oudemansiella platyphylla	74	0	0	0
Panus rudis	0	1	0	0
Phellinus gilvus	0	16	0	0
Phellinus pini	0	8	0	0
Pholiota nameko	8	0	0	0
Pleurocybella porrigens	0	1	0	0
Pleurotus cystidiosus	4	0	0	0
Pluteus cervinus	0	104	0	0
Pluteus longistriatus	12	0	0	0
Pluteus pantherinus	0	7	0	0
Polyporus alveolaris	0	1	0	0
Polyporus elegans	7	0	0	0
Polystictus membranaceus	1	0	0	0
Porodisculus pendulus	1	24	0	0
Psathvrella spadiceogrisea	0	3	0	0
Psathyrella campestris	0	0	0	1
Psathyrella candolleana	2	0	0	0
Psathyrella subinceta	10	0	0	0
Psathyrella velutina	21	0	0	2
Pycnoporus cinnabarinus	13	0	0	0
Pycnoporus coccineus	0	9	0	0
Ramaria botrytoides	1	0	0	0
Ramaria fumigata	0	12	0	3
Ramaria obtusissima	1	0	0	0
Rhodophyllus clypeatus	6	0	0	0
Rhodophyllus lazulinus	0	1	0	0
Rhodophyllus prunuloides	2	0	0	0
Rhodophyllus rhodopolius	17	0	0	0
Russula albida	1	0	0	0
Russula aquosa	85	0	0	0
Russula aurata	8	0	0	0

Russula compacta	7	0	0	0
Russula cyanoxantha	3	0	0	0
Russula decipiens	38	0	0	0
Russula decolorans	28	0	0	0
Russula delica	64	0	0	0
Russula flavida	9	0	0	0
Russula foetens	16	0	0	0
Russula heterophylla	37	0	0	0
Russula luteolacta	2	0	0	0
Russula nigricans	1	0	0	0
Russula olivacea	2	0	0	0
Russula pectinaoides	12	0	0	0
Russula rosea	26	0	0	0
Russula rubra	4	0	0	0
Russula sororia	32	0	0	0
Russula vesca	1	0	0	0
Russula vinosa	82	0	0	0
Russula violeipes	1	0	0	0
Russula virescens	2	0	0	0
Scleroderma areolatum	0	108	0	0
Scutellinia scutellata	0	4	0	0
Sirobasidium magnum	0	1	0	26
Steccherinum ochraceum	0	1	0	0
Stereopsis burtianum	0	1	0	0
Stereum gausapatum	0	5	0	0
Stereum ostrea	0	1	0	0
Stereum rugosum	0	1	0	0
Strobilomyces confusus	1	0	0	0
Strobilomyces seminudus	1	0	0	0
Strobilurus strobilaceus	4	0	0	0
Suillus luteus	10	0	0	0
Termitomyces albuminosus	9	0	0	0
Thelephora aurantiotincta	4	0	0	0
Thelephora caryophellea	0	4	0	0
Trametes gibbosa	1	0	0	0
Trametes griseodura	0	8	0	0
Trametes orientalis	0	2	0	0
Tricholoma flavovirens	0	3	0	0
Tricholoma matsutake	1	0	0	0
Tricholoma terreum	2	0	0	0
Tricholomopsis platyphylla	12	0	0	0
Tylopilus felleus	17	0	0	0

Tylopilus plumbeoviolaceus	2	0	0	0
Tylopilus virens	11	0	0	0
Tyromyces chioneus	0	1	0	0
Volvariella bombycina	11	5	0	0
Volvariella pusilla	14	0	0	0
Volvariella speciosa	0	2	0	0
Volvariella volvacea	26	0	0	0
Xerocomus badius	8	0	0	0
Xeromphalina tenuipes	16	7	0	0
